# Rational design of polymer-based absorbents: application to the fermentation inhibitor furfural

**DOI:** 10.1186/s13068-015-0254-7

**Published:** 2015-05-01

**Authors:** Ikechukwu C Nwaneshiudu, Daniel T Schwartz

**Affiliations:** Department of Chemical Engineering, University of Washington, Box 351750, Seattle, WA 98195-1750 USA

## Abstract

**Background:**

Reducing the amount of water-soluble fermentation inhibitors like furfural is critical for downstream bio-processing steps to biofuels. A theoretical approach for tailoring absorption polymers to reduce these pretreatment contaminants would be useful for optimal bioprocess design.

**Results:**

Experiments were performed to measure aqueous furfural partitioning into polymer resins of 5 bisphenol A diglycidyl ether (epoxy) and polydimethylsiloxane (PDMS). Experimentally measured partitioning of furfural between water and PDMS, the more hydrophobic polymer, showed poor performance, with the logarithm of PDMS-to-water partition coefficient falling between −0.62 and −0.24 (95% confidence). In contrast, the fast setting epoxy was found to effectively partition furfural with the logarithm of the epoxy-to-water partition coefficient falling between 0.41 and 0.81 (95% confidence). Flory-Huggins theory is used to predict the partitioning of furfural into diverse polymer absorbents and is useful for predicting these results.

**Conclusion:**

We show that Flory-Huggins theory can be adapted to guide the selection of polymer adsorbents for the separation of low molecular weight organic species from aqueous solutions. This work lays the groundwork for the general design of polymers for the separation of a wide range of inhibitory compounds in biomass pretreatment streams.

## Introduction

Pretreatment of biomass is a critical step in the biochemical route to low-carbon liquid transportation fuels. Steam explosion, ammonia fiber expansion (AFEX), and dilute acid treatments are frequently used to breakdown and/or reorganize lignin structures in biomass, making sugars more accessible for downstream saccharification and fermentation processes [1-5]. The resulting pretreated biomass stream contains major components (sugar, lignin, and so on) and minor components like organic extractives, water-soluble low molecular weight compounds such as furfural, hydroxymethylfurfural (HMF), and vanillin, as well as other larger degradation products [2]. The water-soluble components are often fermentation inhibitors, so it is desirable to reduce their production or separate them before subsequent bio-processing steps [6].

Several methods are used to remove inhibitory compounds from fermentation broths. Chemical methods can be used to react inhibitory compounds into inactive forms [3,6]. Biological techniques can also be used to degrade furfural and HMF into more benign molecules [7]. Separations using polymers, ion exchange resins, and activated carbon are also an option for inhibitor removal from the pre-fermentation broth [8-12]. For example, the selective removal of furfural from batch solutions has been demonstrated using polystyrene and methacrylic ester resins (Amberlite-based polymers XAD-4, XAD-7) without affecting sugar yields, thereby improving ethanol production in downstream fermentation [8]. Although properties such as column void volume and flow velocity are known to affect the separation, polymer hydrophobicity is the primary factor affecting the performance of both polymer resins [8].

A key thermodynamic parameter that describes the potential of a polymer to separate a target molecule is the partition coefficient [13-18]. Tailoring the traits of the polymer to the trace aqueous component is a common strategy for environmental contaminants like toluene and benzene, where hydrophobic polymers are used to extract low-solubility non-polar molecules from the aqueous solvent [16,19,20]. In contrast, polar fermentation inhibitors like furfural in the pre-fermentation broths will require a more polar polymeric species for an effective separation scheme. The purpose of this paper is to convert chemical intuition-based arguments into a practical theory for guiding polymer selection for optimal absorption-based separation schemes.

The critical parameter that quantifies the absorption partitioning for a species (*i*) between a polymer (*p*) and a solvent (water = *w*) is the equilibrium partition coefficient (${K}_{p/w}^i$), which can be expressed experimentally by [16];

1 $$ {K}_{p/w}^i=\frac{C_p}{C_w} $$

where *C*_*p*_ is the equilibrium concentration of the target compound in the polymer phase and *C*_*w*_ is its equilibrium concentration in the water phase. The larger the ${K}_{p/w}^i$ value, the greater the affinity the target molecule has for the chosen polymer phase. Equilibrium partitioning experiments are routinely performed to determine ${K}_{p/w}^i$ values, but a thermodynamic approach suitable for prediction of trace aqueous components will be a key advancement in the selection of the optimal polymer for use in separations. Several approaches to predicting the partition coefficient can be found in the literature. These include molecular connectivity index [21], linear solvation energy relationships (LSERs) [22], and Flory-Huggins theory [23-25]. In our situation, using a Flory-Huggins theoretical approximation is appropriate because it accounts for size disparities between aqueous solute and polymer [24,26,27] and can be further simplified for dilute, low molecular weight contaminants partitioning into large polymer chains, resulting in the expression:

2 $$ \log \left({K}_{p/w}^i\right)=- \log \left({S}_w^i{\overline{V}}_i\right)-\frac{\left[1+\chi \right]}{2.303} $$

where ${K}_{p/w}^i$ is the equilibrium partition coefficient of the target molecule (*i*) between polymer (*p*) and water (*w*) phases, ${S}_w^i$ and ${\overline{V}}_i$ are the water solubility and molar volume of the molecule (*i*) being extracted. The Flory-Huggins interaction parameter *χ* can be further approximated by using the molecule and polymer solubility parameters, *δ*_*i*_ and *δ*_*p*_, respectively:

3 $$ \chi =\frac{{\left({\delta}_i-{\delta}_p\right)}^2{\overline{V}}_i}{\mathrm{RT}} $$

where *R* is the universal gas constant and *T* is temperature (K). Key simplifying assumptions in Equation 2 are the following: 1) ${\overline{V}}_i/{\overline{V}}_p\to\ 0$ (molar volume of molecule is significantly smaller than that of the polymer), and the trace contaminant is dilute in both the solution and polymer. Here, we show the use of the simple set of Equations 2 and 3 as the basis for guiding the selection of polymer for the targeted inhibitor furfural found in most biomass pretreatment broths.

## Materials and methods

### Sample preparation

#### PDMS samples

Slygard 184 and catalyst for making PDMS were obtained from Dow Corning (Midland, MI, USA). Samples of PDMS used in the experiment were prepared as follows. Uncured PDMS was mixed using the standard 10:1 polymer to catalyst ratio in a poly(methyl-methacrylate) (PMMA) mold 1.5 mm deep. This was cured at 70°C overnight to make an approximately 1.5-mm-thick sheet of PDMS. Samples were punched out of the PDMS sheet using a 2-mm diameter punch. The resulting PDMS plugs were sealed and kept until used.

#### Epoxy samples

Fast-curing epoxy (bisphenol A diglycidyl ether resin) was obtained from ITW Devcon (Danvers, MA, USA). Epoxy resin and hardener were poured and mixed on a sheet of paper and spread into a thin layer approximately 1 to 2 mm thick. This was cured at room temperature for 4 h, allowing the polymer resin to harden completely. Samples approximately 2 × 4 mm samples were cut out of the slab using a razor blade. Epoxy slabs were sealed and kept until used.

#### Organic/aqueous solutions

Furfural was purchased from Sigma-Aldrich (St. Louis, MO, USA) and used as received. Experimental solutions were prepared by adding the appropriate amount of furfural into 20 mL of deionized (DI) water. The solutions were mixed, tightly capped, and left to sit until fully dissolved. These solutions were used within 15 min of preparation.

### Polymer absorption procedure

Furfural solutions in 20-mL glass scintillation vials were used for the absorption/partitioning procedure. Three sample plugs (PDMS) or slabs (epoxy) were introduced to each vial, tightly capped, and left to equilibrate undisturbed at room temperature for more than 48 h. The final concentration in the aqueous solution after equilibration is

4 $$ {C}_f={C}_o\frac{1}{1+K\left({V}_p/{V}_s\right)} $$

where *C*_*f*_ and *C*_*o*_ are the final and initial concentrations in the liquid sample (respectively), *V*_*p*_ and *V*_*s*_ are the volumes of the polymer and liquid sample (respectively), and *K* is the partition coefficient. From this equation, we see, due to the small size of our polymer pieces and scale of *K* (determined later), that there is negligible change in concentration after equilibration with the polymer.

### Raman spectra acquisition

Spectra were collected using a Renishaw inVia Raman micro-spectrometer (Renishaw, Wotton-under-Edge, UK) attached to a Leica DM IRBE upright optical microscope (Leica, Wetzlar, Germany). A 785-nm diode laser operated at full power (nominal 180 mW) was used to irradiate samples through a 50× (N.A. 0.8) objective lens. The spot area was approximately 50 μm [2]. Scattered light was acquired through the same objective lens and detected on a thermoelectrically cooled (−60°C) CCD. Spectra were typically acquired for 10 s at 100% laser power, except where fluorescence was an issue. For example, spectra for the equilibrated epoxy samples were collected using 100 acquisitions of 1-s duration to avoid saturation of the detector from fluorescence. The spectrum of furfural (as received) was acquired at 1% of the nominal laser power due to the fluorescence of the sample. All sample spectra were collected using a wet sample holder sealed with an optical coverslip. Laser stability was assured using the 520 cm^−1^ peak of silicon as a reference. The PDMS peak at 1,410 cm^-1^ and the epoxy peak at 1,610 cm^-1^ was acquired with all polymer phase spectra and served as an internal standard that helped normalize for sample to sample variations in optical focus.

### Raman peak normalization and data analysis

Spectral peaks were analyzed using Wire 2.0 software. All spectra were baseline subtracted using second-order polynomial or cubic spline functions, producing very flat baselines for subsequent analysis. Intensities were normalized by total acquisition time. Spectral peaks where fit to standard Voigt distribution profiles. Curve fit parameters were used to calculate the reported integrated peak areas. Peak areas are reported in counts per second (cps). Normalized peak intensities were analyzed in the R Statistical Software package to for the linear regression analysis and determination of 95% confidence intervals for all parameters reported here [http://www.r-project.org/].

## Results and discussion

**Figure 1:**
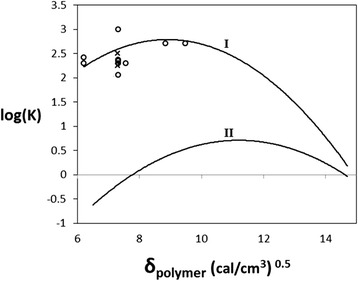
Flory-Huggins theoretical estimations. Flory-Huggins theoretical estimations of polymer/toluene (I) and polymer/furfural (II) partition coefficients (curves) for a realistic range of polymer solubility parameters (6 ≤ *δ*_polymer_ ≤ 14.5). Experimental measurements for toluene partitioning into five different polymers is found in our prior published work (*x*) and results from other groups (*o*).

To the best of our knowledge, the simplifications of Flory-Huggins theory that result in Equations 2 and 3 have not been evaluated for absorption partitioning. We assess the reasonableness of this Flory-Huggins approximation for predicting polymer/compound partition coefficients by using widely reported experimental data for the aqueous partition of toluene into various polymer phases. Figure 1 (I) shows the partition coefficient predictions, $\log \left({K}_{p/w}^i\right)$, calculated from Equations 2 and 3 for toluene as a function of polymer solubility parameter *δ*_*p*_ over the range represented by the polymers listed in Table 1. Here, we use a toluene solubility parameter of *δ*_*i*_ = 8.9, an aqueous solubility of ${S}_w^{\mathrm{Toluene}} = 5.1\ \mathrm{m}\mathrm{M}$ at 25°C, and a molar volume of ${\overline{V}}_{\mathrm{Toluene}} = 106.3\ \mathrm{mL}/\mathrm{mol}$. The data in Table 1 shows that polymers such as PDMS (*δ*_*p*_ = 7.3), polystyrene-co-butadiene (PSB, *δ*_*p*_ = 8.84), and polyacrylonitrile-co-butadiene (PAB, *δ*_*p*_ = 9.48) are close to the theoretical maximum log (${K}_{p/w}^i$) for toluene.

**Table 1:** **Solubility parameters of different polymers we use to link theory to actual polymers of interest**

**Polymers**	**(** ***δ*** **= (cal/cm** ^**3**^ **)** ^**0.5**^
Poly(tetrafluoroethylene)	6.50
Poly(dimethylsiloxane)	7.30
Poly(butadiene)	7.57
Poly(ethylene)	8.00
Poly(propylene)	8.20
Poly(n-butyl methacrylate)	8.70
Poly(ethyl methacrylate)	9.04
Poly(n-butyl acrylate)	9.04
Poly(chloroprene)	9.04
Poly(styrene)	9.13
Poly(phenyloxide)	9.15
Poly(methyl methacrylate)	9.30
Acrylic	9.40
Poly(vinyl chloride)	9.50
Poly(vinyl acetate)	9.80
Poly(methyl acrylate)	10.02
Poly(ethylene terephthalate)	10.10
Bisphenol A epoxy resin	10.70
Cellulose acetate	11.70
Nylon 6,6	13.70
Poly(ethylene oxide)	14.70

Data in table extracted from [21].

Also included in Figure 1 are experimental values of $\log \left({K}_{p/w}^{\mathrm{Toluene}}\right)$ found in the literature for the partitioning of toluene into five different polymer phases. Symbols *x* and *o* represent experimental values from our work [16] and literature sources [28-31], respectively. From these data, we can see that the Flory-Huggins theoretical predictions adequately estimate the partition coefficient of toluene across several different polymer extraction phases. There is significant variation in the experimentally derived PDMS/toluene partition coefficients (Figure 1). We can only hypothesize why this is. The measurements come from a wide range of different experimental methods, including MIMS (membrane inlet mass spectrometry), Raman spectroscopy, UV-Vis spectroscopy, and IR spectroscopy. Nominally, to be reported as a partition coefficient, each method assumed equilibrium partitioning had been achieved between the PDMS phase and the aqueous toluene sample. Moreover, when we place each point at identical x-axis locations, we assume all PDMS polymers were identical. It is challenging to know exactly how these fundamental assumptions may interact to drive the wide variation in reported values for the partition coefficient for toluene in PDMS.

In the case of the fermentation inhibitor furfural, we also generate a similar theoretically curve to optimize partitioning and absorption using a furfural’s solubility parameter *δi* = 11.2 [24], an aqueous solubility of ${S}_w^{\mathrm{Furfural}} = 865\ \mathrm{m}\mathrm{M}$, and a molar volume of ${\overline{V}}_{\mathrm{Furfural}} = 83.2\ \mathrm{mL}/\mathrm{mol}$. Figure 1 (II) shows the predicted furfural partition coefficient between the polymer and water phases as log (${K}_{p/w}^{\mathrm{Furfural}}$) versus polymer solubility parameter. Compared to toluene, the theoretical maximum log (${K}_{p/w}^i$) for furfural is significantly lower due to its greater water solubility. Figure 1 (II) also displays a diverse range of possible log (${K}_{p/w}^{\mathrm{Furfural}}$) values from negative to positive, which shows the optimum range of polymer solubility parameters is 10 ≤ *δ*_*p*_ ≤ 12.5. Using this plot and Table 1, we can find the polymer phases that likely give the maximum furfural partitioning.

To experimentally demonstrate the robustness of our approach, we select a high-performing and low-performing polymer absorption phase using the data in Figure 1 (II). Fast-curing epoxy and PDMS are both cross-linking polymer networks with significantly different solubility parameters. PDMS, a conventional absorption polymer used often in analytical chemistry techniques, has a solubility parameter *δ*_*p*_ = 7.3. Epoxy, a polymeric resin most widely used as an adhesive, has a solubility parameter *δ*_*p*_ = 10.7. The Flory-Huggins approximation shows that for furfural, PDMS polymer should display less favorable partitioning with a negative log (${K}_{p/w}^i$) value, while epoxy should have enhanced partitioning with a positive log (${K}_{p/w}^i$) that is close to the theoretical maximum.

**Figure 2:**
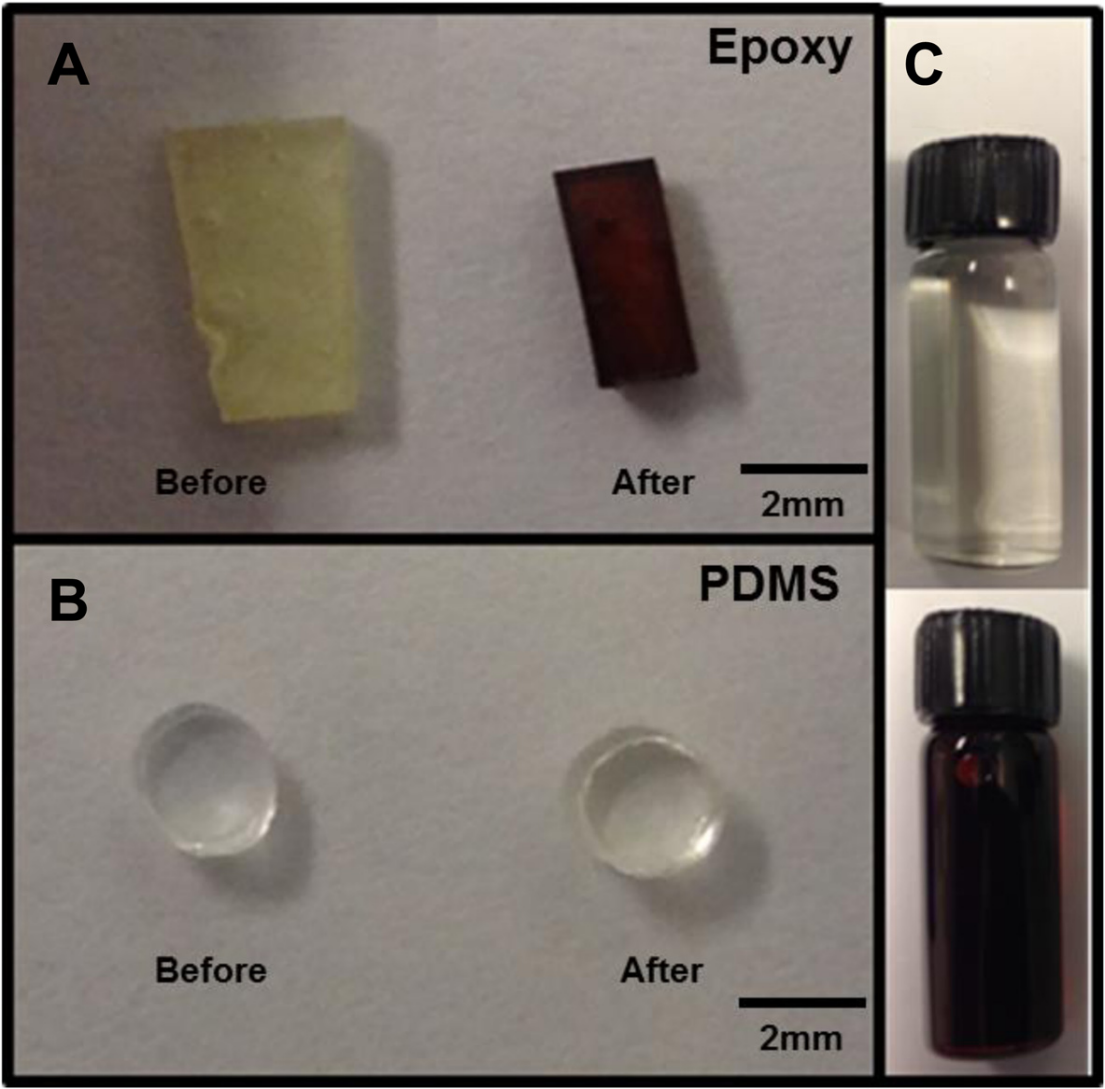
Two slabs of epoxy, two plugs of PDMS, and two vials of furfural. **(A)** Two slabs of epoxy before and after equilibration in 50 mM Furfural. **(B)** Two plugs of PDMS before and after equilibration in 50 mM Furfural. **(C)** Top 2-ml vial is filled with 50 mM furfural and the bottom is filled with furfural (as received).

The two polymers were equilibrated in a 50-mM solution of furfural and water. Figure 2 is a visual illustration of the equilibrium partitioning of furfural from solution into the two polymer phases. Figure 2A shows pieces of epoxy resin (one before (left) and the other (right) after being introduce to 50-mM solution of furfural), while Figure 2B shows pieces of PDMS before and after the same equilibration. Figure 2C shows the color of 50-mM furfural solution (top) and furfural as received (bottom) contained in 2-ml glass vials. The as-received furfural (99% pure) was dark brown in color, even though pure furfural should be clear. The brown color in stored furfural is caused by acidic impurities and resins produced when furfural autoxidizes [32]. For example, 5-methylfurfural is a brown- colored impurity. Figure 2A shows that these impurities seem to partition strongly into the epoxy phase causing the color change from yellow (before) to brown (after) equilibration. However, the change seen in PDMS, before and after equilibration (B), is not as drastic. The strongly colored epoxy sample produced strong fluorescence during Raman spectral acquisition.

**Figure 3:**
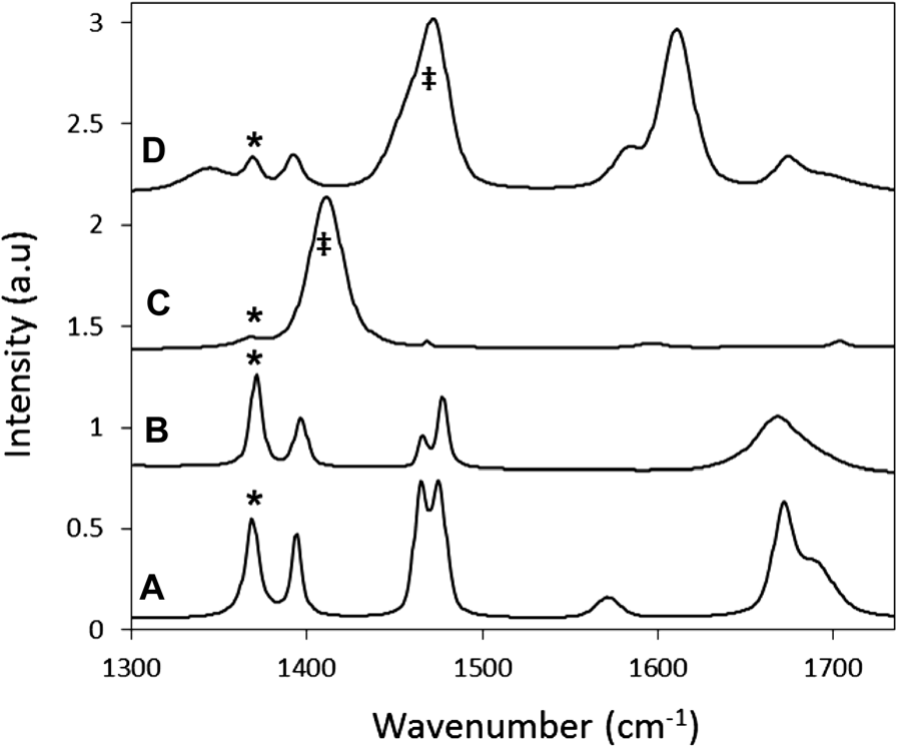
Fingerprinting Raman spectra of furfural in different phases. **(A)** Furfural (as received), **(B)** dissolved in water, **(C)** partitioned from water into PDMS, **(D)** partitioned from water into Epoxy. Asterisks (*) indicate furfural spectral peak used for analysis. (‡) indicate internal standards.

Raman fingerprinting scans show that the 1,372 cm^−1^ peak for the fermentation inhibitor furfural can be clearly discerned in all experimental polymer and solvent phases, even with a fluorescence impurity in the as received furfural. Figure 3 shows characteristic Raman spectra of furfural in four different solvent/polymer environments within the 1,300 to 1,750 cm^−1^ wavenumber window. Spectrum A shows the characteristic peaks of furfural (as-received) while spectrum B shows it dissolved in water. Spectra C and D are from the epoxy and PDMS polymers, respectively, equilibrated in aqueous furfural. The fingerprint scans show that the 1,372 cm^−1^ furfural peak is distinct from the background polymer and water phases, and each polymer phase has a strong background signal that can be used as an internal standard for peak intensity normalization.

**Figure 4:**
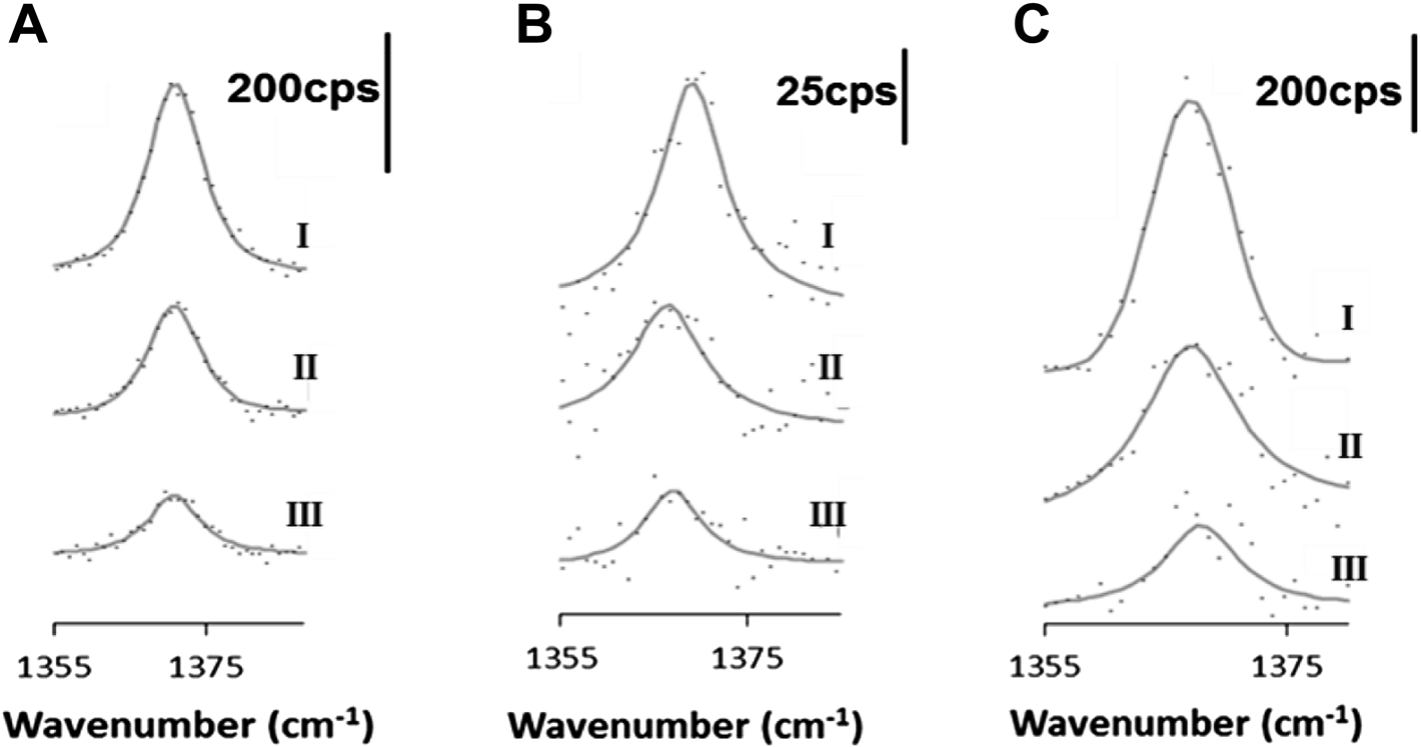
Raman spectra of the 1,372 cm^−1^ furfural peak. Panels **(A)**, **(B)**, and **(C)** are Raman spectra of the 1,372 cm^−1^ furfural peak acquired in the aqueous phase and the equilibrated PDMS and epoxy phases, respectively. Three different aqueous concentrations are shown: (I) 50 mM, (II) 35 mM, and (III) 20 mM. Data points are raw data, and lines are the Voigt curve fits. The normalize signal intensity is shown as counts per second (cps).

To quantify partitioning, we do more careful fitting of the normalized furfural peak intensities as a function of aqueous furfural concentration in each phase being studied. Figure 4A shows the aqueous 1,372 cm^−1^ furfural peak, as dissolved in water at 50-, 35-, and 20-mM concentrations (curves I, II, and III, respectively). Figure 4B shows the same furfural peak in PDMS equilibrated at the same aqueous concentrations, again denoted, I, II, and III. The furfural peak location is slightly shifted due to the change in chemical environment. Figure 4C shows the furfural peak taken in the epoxy equilibrated with the aqueous furfural (also at identical concentrations, I, II, and III). The baseline-subtracted spectral data points are presented along with corresponding curve fits, and scale bars show the intensity of the Raman signal in counts per second (cps). Firstly, we see that the signal acquired in epoxy is enhanced compared to the water phase. We also see that the signal in PDMS is significantly attenuated compared to the water phase. It is evident (qualitatively) that aqueous furfural partitions preferentially in epoxy compared to PDMS. The range of furfural concentrations used here represents a balance between maximizing Raman signal (which favors high concentrations) and a desire to meet the constraints of our polymer absorption theory, namely, dilute solute in the polymer phases. Reported furfural concentrations in fermentation broths can be in the range 2 to 5 g/L which is exactly the range used here [5,33].

**Figure 5:**
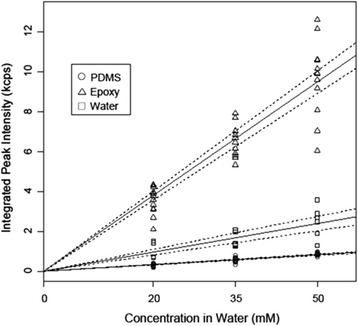
Integrated peak intensity vs. water concentration plot. Replicate measurements for the integrated 1,372 cm^−1^ furfural Raman peak intensities are shown as a function of the concentrations in the water phase. Linear fits (solid lines) and 95% confidence intervals (dashed lines) are presented for the Raman signals acquired in the equilibrated epoxy phase (triangle), equilibrated PDMS phase (circle), and the water phase (square).

We quantify aqueous partitioning of furfural into both PDMS and epoxy phases by using the method detailed in prior work, though here we go more deeply into sampling methods that let us better estimate measurement uncertainty [16,20]. Figure 5 is a plot of the entire normalized dataset, with the linear regression fits (solid line) and corresponding 95% confidence (dashed) intervals (dashes) in epoxy (triangles), water (squares), and PDMS (circles) phases. The confidence intervals were generated using the R statistical software package. Replicate data points at each concentration are from six different spectra acquired at two different microscopic locations (per sample) on three equilibrated samples (two different trials). Peak areas were normalized to a PDMS or epoxy internal standard peaks at 1,410 or 1,610 cm^−1^, respectively (Figure 3). This reduces variation based on solid phase sample preparation so that peaks can be directly related to furfural concentration (assuming negligible polymer swelling by the solvent or solute). We estimate the furfural partition coefficient between water and polymer ${K}_{\mathrm{Polymer}/\mathrm{Water}}^{\mathrm{Furfural}}$ from the data in Figure 5 as:

5 $$ {K}_{p/w}^{\mathrm{Furfural}} = \frac{{\left(\mathrm{Slope}\right)}_{p/w}}{{\left(\mathrm{Slope}\right)}_w} $$

where (Slope)_*p*/*w*_ is the slope of the fit for furfural measured directly in either epoxy or PDMS phase in contact with the aqueous solution, and (Slope)_*w*_ is the slope of the best fit line measured directly in the aqueous phase. Based on the best fit slopes, the mean value of aqueous furfural partitioning into PDMS is $\log \left({K}_{\mathrm{PDMS}/w}^{\mathrm{Furfural}}\right)$ = −0.47 and into epoxy is $\log \left({K}_{\mathrm{Epoxy}/w}^{\mathrm{Furfural}}\right)$ = 0.59. The 95% confidence intervals for the furfural partition coefficient between PDMS and water is −0.62 ≤ $log\left({K}_{\mathrm{PDMS}/w}^{\mathrm{Furfural}}\right)$ ≤ −0.24 and for epoxy and water it is 0.41 ≤ $\log \left({K}_{\mathrm{Epoxy}/w}^{\mathrm{Furfural}}\right)$ ≤ 0.81. We estimated these 95% confidence intervals by random sampling with replacement using the boot strapping method implemented in the R statistical software package. For the results presented here, the data was randomly sampled 1,000 times. Table 2 shows the experimentally derived and predicted values of log *K* of both polymers in water. We see that our values match the predicted trend, in that PDMS gives a negative value of $\log \left({K}_{\mathrm{PDMS}/w}^{\mathrm{Furfural}}\right)$, while fast-curing epoxy gives a positive $\log \left({K}_{\mathrm{Epoxy}/w}^{\mathrm{Furfural}}\right)$ value as predicted by the Flory-Huggins approximations.

**Table 2:** **Experimental values of log(** ***K***_***f***_ **)**

**SPME polymer phases**	**Log(** ***K***_***f***_ **)**	**Log(** ***K***_***f***_ **)**
	**−λ ≤ Mean ≤ +λ**	**(Flory-Huggins)**
Epoxy	0.41 ≤ 0.59 ≤ 0.81	0.70
PDMS	−0.62 ≤ −0.47 ≤ −0.24	−0.21

Experimental values of log(*Kf*), including the mean and 95% confidence range (±λ), for partitioning from water into epoxy and PDMS are compared to the Flory-Huggins approximation.

### Conclusions and implications

We show the general effectiveness of using Flory-Huggins theory for screening polymer materials to use as solid phase absorbents for dilute aqueous solutes. Experimentally and theoretically, we show that PDMS has unfavorable partitioning of furfural from water, whereas epoxy has favorable partitioning. Flory-Huggins theory shows that, in the simple dilute limit we explored, the two main factors affecting the separation are the solubility of the solute in water and how closely matched the polymer-furfural solubility parameters were. Using data in the literature for toluene, we showed that this approach is reasonable for other solutes than furfural, and we believe it is a general approach that is applicable to other compounds present in pre-fermentation broths.

Our simplified Flory-Huggins approach has implications for bioprocess design. First, in a dilute mixture with multiple inhibitors, the partitioning calculation, to first approximation, is independent for each inhibitor species. That means optimal materials can be selected fairly easily. At the same time, selectivity (that is, desire to separate inhibitory solutes and not sugars) can also be assessed based on this theory, by looking for polymers that maximize inhibitor partitioning and minimize sugar partitioning. Secondarily, this work has implications for process design. Equation 4 describes the reduction in inhibitor compound concentration when a batch of pre-fermentation broth is contacted with a fresh absorbent polymer. Using furfural absorption into an epoxy resin as an example, we see that Equation 4 predicts a 50% reduction in furfural concentration from a single equilibrium stage when the polymer-to-solution volume ratio is approximately 1:4, since K ≈ 4. Of course, one can design packed beds with multiple equilibrium stages to get higher separation factors. Bed regeneration can be carried out using clean water. Separation design based on cyclic operation that alternates between a regeneration loop and absorption loop can be conceived, though it is beyond the scope of this paper. Of course, accurate equilibrium stage calculations may require a more comprehensive thermodynamic model for partitioning into the solid phase, especially to accommodate the high concentrated sugar species, as our main purpose was to guide materials selection.

## Abbreviations

AFEX: ammonia fiber expansion
cps: counts per second
DI: deionized
Epoxy/fast-curing epoxy: 5 bisphenol A diglycidyl ether
HMF: hydroxymethylfurfural
LSERs: linear solvation energy relationships
PAB: polyacrylonitrile-co-butadiene
PDMS: poly(dimethylsiloxane)
PMMA: poly(methyl-methacrylate)
PSB: polystyrene-co-butadiene
